# (*E*)-4-Amino-*N*′-(2-hy­droxy-5-meth­oxy­benzyl­idene)benzohydrazide monohydrate

**DOI:** 10.1107/S1600536812026633

**Published:** 2012-07-04

**Authors:** Hadi Kargar, Reza Kia, Muhammad Nawaz Tahir

**Affiliations:** a Department of Chemistry, Payame Noor University, PO Box 19395-3697 Tehran, I. R. of IRAN; b Department of Chemistry, Science and Research Branch, Islamic Azad University, Tehran, Iran; c Department of Physics, University of Sargodha, Punjab, Pakistan

## Abstract

In the title compound, C_15_H_15_N_3_O_3_·H_2_O, the hydazide Schiff base mol­ecule shows an *E* conformation around the C=N bond. An intra­molecular O—H⋯N hydrogen bond makes an *S*(6) ring motif. The dihedral angle between the substituted phenyl rings is 23.40 (11)°. The water mol­ecule mediates linking of neighbouring mol­ecules through O—H⋯(O,O) hydrogen bonds into infinite chains along the *a* axis, which are further connected together through N—H⋯O hydrogen bonds, forming a two-dimensional network parallel to (001). C—H⋯O inter­actions aso occur.

## Related literature

For standard bond lengths, see: Allen *et al.* (1987 ▶). For hydrogen-bond motifs, see: Bernstein *et al.* (1995 ▶). For the coordination chemistry of Schiff base and hydrazone derivatives, see: Kucukguzel *et al.* (2006 ▶); Karthikeyan *et al.* (2006 ▶). For 4-amino­benzohydrazide-derived Schiff base structures, see: Xu (2012 ▶); Shi & Li (2012 ▶); Bakir & Green (2002 ▶); Kargar *et al.* (2012**a* ▶,b* ▶). 
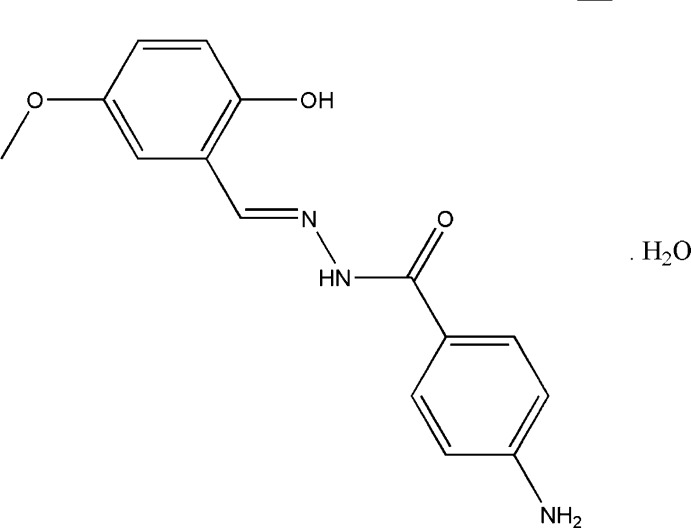


## Experimental

### 

#### Crystal data

C_15_H_15_N_3_O_3_·H_2_O
*M* *_r_* = 303.32
Monoclinic, 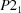

*a* = 4.7376 (5) Å
*b* = 13.270 (2) Å
*c* = 11.7265 (16) Å
β = 98.459 (4)°
*V* = 729.18 (17) Å^3^
*Z* = 2
Mo *K*α radiation
μ = 0.10 mm^−1^
*T* = 291 K
0.28 × 0.20 × 0.18 mm

#### Data collection

Bruker SMART APEXII CCD area-detector diffractometer
Absorption correction: multi-scan (*SADABS*; Bruker, 2005 ▶) *T*_min_ = 0.972, *T*_max_ = 0.982
6511 measured reflections
1679 independent reflections
1433 reflections with *I* > 2σ(*I*)
*R*_int_ = 0.028

#### Refinement

*R*[*F* ^2^ > 2σ(*F* ^2^)] = 0.035
*wR*(*F* ^2^) = 0.085
*S* = 1.03
1679 reflections
200 parameters
1 restraint
H-atom parameters constrained
Δρ_max_ = 0.14 e Å^−3^
Δρ_min_ = −0.13 e Å^−3^

### 

Data collection: *APEX2* (Bruker, 2005 ▶); cell refinement: *SAINT* (Bruker, 2005 ▶); data reduction: *SAINT*; program(s) used to solve structure: *SHELXS97* (Sheldrick, 2008 ▶); program(s) used to refine structure: *SHELXL97* (Sheldrick, 2008 ▶); molecular graphics: *SHELXTL* (Sheldrick, 2008 ▶)’; software used to prepare material for publication: *SHELXTL* and *PLATON* (Spek, 2009 ▶).

## Supplementary Material

Crystal structure: contains datablock(s) global, I. DOI: 10.1107/S1600536812026633/bq2366sup1.cif

Structure factors: contains datablock(s) I. DOI: 10.1107/S1600536812026633/bq2366Isup2.hkl

Supplementary material file. DOI: 10.1107/S1600536812026633/bq2366Isup3.cml

Additional supplementary materials:  crystallographic information; 3D view; checkCIF report

## Figures and Tables

**Table 1:** Hydrogen-bond geometry (Å, °)

*D*—H⋯*A*	*D*—H	H⋯*A*	*D*⋯*A*	*D*—H⋯*A*
O1*W*—H1*W*1⋯O1^i^	0.92	2.00	2.926 (3)	174
O2—H2⋯N3	0.93	1.85	2.650 (3)	143
O1*W*—H2*W*1⋯O1^ii^	0.83	1.95	2.787 (3)	176
N2—H2*N*⋯O1*W*	0.95	2.15	3.084 (3)	167
N1—H1*N*1⋯O3^iii^	0.93	2.25	3.043 (3)	143
N1—H2*N*1⋯O2^i^	0.99	2.17	3.141 (3)	169
C2—H2*A*⋯O1*W*	0.93	2.45	3.351 (3)	163
C8—H8*A*⋯O1*W*	0.93	2.56	3.368 (3)	146

Symmetry codes: (i) 
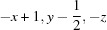
; (ii) 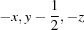; (iii) 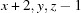.

**Fig. 1.**
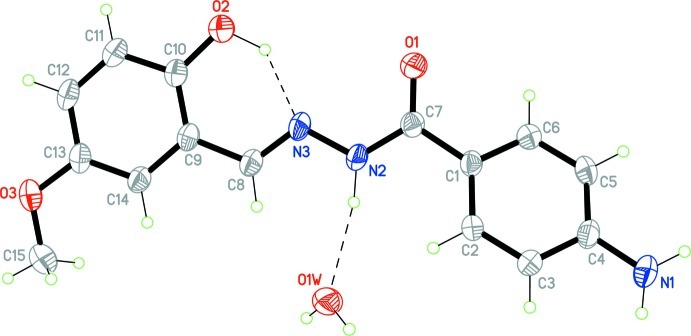
A view of the molecular structure of the title compound, showing 40% probability displacement ellipsoids and the atomic numbering. The dashed lines shows the intramolecular hydrogen bonds.

**Fig. 2.**
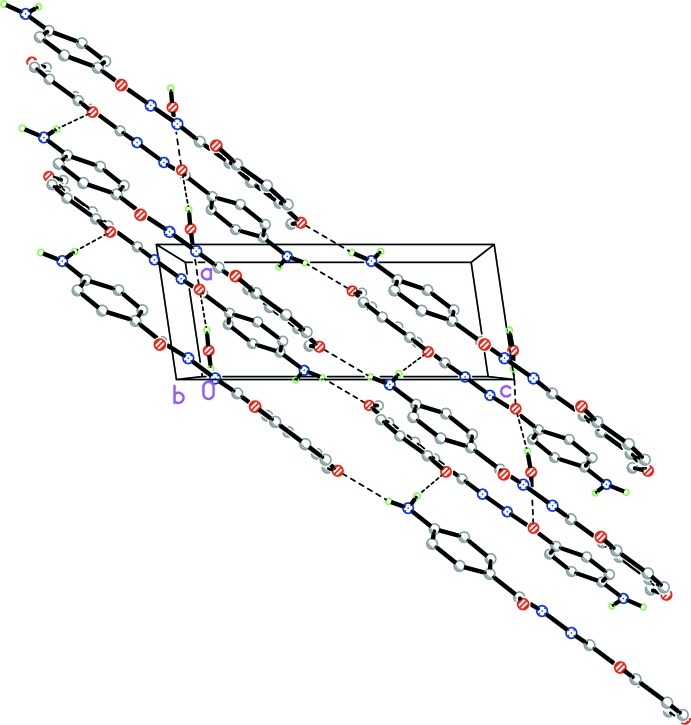
A view along the a axis of crystal packing of the title compound, showing linking of molecules through the intermolecular N—H···O and O—H···O interactions (dashed lines), forming two-dimensional networks. Only the H atoms involved in the interactions are shown.

## supplementary crystallographic information

### Comment

Schiff bases are one of the most prevalent mixed-donor ligands in the field of coordination chemistry. They play an important role in the development of coordination chemistry related to catalysis and magnetism, and supramolecular architectures (Karthikeyan *et al.,* 2006; Kucukguzel *et al.,* 2006). Structures of Schiff bases derived from substituted 4-aminobenzohydrazide have been reported earlier (Kargar *et al.,* 2012**a*,b*; Xu, 2012; Shi & Li, 2012; Bakir & Green, 2002). In order to explore the structure of the new Schiff base derivatives, the title compound was prepared and characterized crystallographically.

The asymmetric unit of the title compound, Fig. 1, comprises a molecule of the title hydazide Schiff base and a water molecule of crystallization. It shows *E* conformation around C═N bond. The bond lengths (Allen *et al.*, 1987) and angles are within normal ranges and are comparable to the related structures (Kargar *et al.,* 2012**a*,b*; Xu, 2012; Shi & Li, 2012; Bakir & Green, 2002). Intramolecular O—H···N hydrogen bond makes *S(6)* ring motif (Bernstein *et al.,* 1995). The dihedral angle between the substituted phenyl rings is 23.40 (11)Å. The water molecule mediates linking of the neighboring molecules through O—H···(O, O) hydrogen bondings into infinite chains along the *a* axis which are further connected together through N—H···O hydrogen bonds, forming two-dimensional network parallel to (0 0 1) [Fig. 2].

### Experimental

The title compound was synthesized by adding 1 mmol of methyl 4-aminobenzoate to a solution of 5-methoxysalicylaldehyde (1 mmol) in methanol (30 ml). The mixture was refluxed with stirring for 50 min and after cooling to room temperature a light-yellow precipitate was filtered and washed with diethylether and dried in air. white prismatic crystals of the title compound, suitable for *X*-ray structure analysis, were recrystallized from ethanol by slow evaporation of the solvents at room temperature over several days.

### Refinement

The N- and O-bound H-atoms were located in a difference Fourier map and constrained to refine to the parent atoms with U_iso_ (H) = 1.2 or 1.5 U_eq_(N, O), respectively, see Table 1. The rest of the H atoms were positioned by riding model approximation with C—H = 0.93 and U_iso_ (H) = k × U_eq_(C) with k = 1.2 for CH and 1.5 for CH_3_. In the absence of sufficient anomalous scattering 1437 Friedel pairs were merged.

### Crystal data

**Table:**

C_15_H_15_N_3_O_3_·H_2_O	*F*(000) = 320
*M**_r_* = 303.32	*D*_x_ = 1.381 Mg m^−^^3^
Monoclinic, *P*2_1_	Mo *K*α radiation, λ = 0.71073 Å
Hall symbol: P 2yb	Cell parameters from 873 reflections
*a* = 4.7376 (5) Å	θ = 2.5–28.5°
*b* = 13.270 (2) Å	µ = 0.10 mm^−^^1^
*c* = 11.7265 (16) Å	*T* = 291 K
β = 98.459 (4)°	Prism, white
*V* = 729.18 (17) Å^3^	0.28 × 0.20 × 0.18 mm
*Z* = 2	

### Data collection

**Table:**

Bruker SMART APEXII CCD area-detector diffractometer	1679 independent reflections
Radiation source: fine-focus sealed tube	1433 reflections with *I* > 2σ(*I*)
Graphite monochromator	*R*_int_ = 0.028
φ and ω scans	θ_max_ = 27.2°, θ_min_ = 1.8°
Absorption correction: multi-scan (*SADABS*; Bruker, 2005)	*h* = −5→6
*T*_min_ = 0.972, *T*_max_ = 0.982	*k* = −17→17
6511 measured reflections	*l* = −15→15

### Refinement

**Table:**

Refinement on *F*^2^	Primary atom site location: structure-invariant direct methods
Least-squares matrix: full	Secondary atom site location: difference Fourier map
*R*[*F*^2^ > 2σ(*F*^2^)] = 0.035	Hydrogen site location: inferred from neighbouring sites
*wR*(*F*^2^) = 0.085	H-atom parameters constrained
*S* = 1.03	*w* = 1/[σ^2^(*F*_o_^2^) + (0.0414*P*)^2^ + 0.0579*P*] where *P* = (*F*_o_^2^ + 2*F*_c_^2^)/3
1679 reflections	(Δ/σ)_max_ < 0.001
200 parameters	Δρ_max_ = 0.14 e Å^−^^3^
1 restraint	Δρ_min_ = −0.13 e Å^−^^3^

### Special details

**Table:**

Geometry. All esds (except the esd in the dihedral angle between two l.s. planes) are estimated using the full covariance matrix. The cell esds are taken into account individually in the estimation of esds in distances, angles and torsion angles; correlations between esds in cell parameters are only used when they are defined by crystal symmetry. An approximate (isotropic) treatment of cell esds is used for estimating esds involving l.s. planes.
Refinement. Refinement of F^2^ against ALL reflections. The weighted R-factor wR and goodness of fit S are based on F^2^, conventional R-factors R are based on F, with F set to zero for negative F^2^. The threshold expression of F^2^ > 2sigma(F^2^) is used only for calculating R-factors(gt) etc. and is not relevant to the choice of reflections for refinement. R-factors based on F^2^ are statistically about twice as large as those based on F, and R- factors based on ALL data will be even larger.

### Fractional atomic coordinates and isotropic or equivalent isotropic displacement parameters (Å^2^)

**Table:**

	*x*	*y*	*z*	*U*_iso_*/*U*_eq_	
O1	0.2636 (4)	0.77661 (14)	−0.05541 (17)	0.0508 (5)	
O2	−0.2127 (4)	0.80740 (14)	0.21112 (17)	0.0525 (5)	
H2	−0.1070	0.7823	0.1570	0.079*	
O3	−0.7483 (4)	0.51216 (16)	0.43124 (16)	0.0576 (6)	
O1W	0.2220 (4)	0.39655 (14)	0.06445 (17)	0.0554 (5)	
H1W1	0.3915	0.3618	0.0655	0.083*	
H2W1	0.0724	0.3628	0.0602	0.083*	
N1	0.9460 (5)	0.49031 (19)	−0.3593 (2)	0.0563 (6)	
H1N1	1.0085	0.5262	−0.4186	0.084*	
H2N1	1.0325	0.4284	−0.3229	0.084*	
N2	0.1583 (4)	0.62403 (16)	0.01294 (17)	0.0385 (5)	
H2N	0.1776	0.5530	0.0164	0.046*	
N3	−0.0003 (4)	0.66404 (16)	0.09130 (17)	0.0380 (5)	
C1	0.4541 (4)	0.6311 (2)	−0.1357 (2)	0.0343 (5)	
C2	0.5460 (5)	0.53219 (18)	−0.1196 (2)	0.0389 (6)	
H2A	0.4945	0.4956	−0.0582	0.047*	
C3	0.7107 (5)	0.48700 (19)	−0.1917 (2)	0.0424 (6)	
H3A	0.7718	0.4209	−0.1778	0.051*	
C4	0.7876 (5)	0.5391 (2)	−0.2859 (2)	0.0409 (6)	
C5	0.6937 (6)	0.6372 (2)	−0.3037 (2)	0.0470 (6)	
H5A	0.7407	0.6732	−0.3664	0.056*	
C6	0.5309 (5)	0.6824 (2)	−0.2298 (2)	0.0439 (6)	
H6A	0.4714	0.7487	−0.2432	0.053*	
C7	0.2861 (5)	0.68406 (19)	−0.0575 (2)	0.0359 (5)	
C8	−0.1204 (5)	0.59925 (19)	0.1497 (2)	0.0388 (6)	
H8A	−0.0947	0.5309	0.1370	0.047*	
C9	−0.2965 (5)	0.6304 (2)	0.2356 (2)	0.0357 (5)	
C10	−0.3358 (5)	0.7308 (2)	0.2623 (2)	0.0386 (5)	
C11	−0.5064 (5)	0.7548 (2)	0.3461 (2)	0.0462 (7)	
H11A	−0.5315	0.8219	0.3651	0.055*	
C12	−0.6366 (6)	0.6808 (2)	0.4005 (2)	0.0459 (7)	
H12A	−0.7489	0.6981	0.4562	0.055*	
C13	−0.6028 (5)	0.5804 (2)	0.3732 (2)	0.0421 (6)	
C14	−0.4317 (5)	0.5551 (2)	0.2919 (2)	0.0404 (6)	
H14A	−0.4058	0.4877	0.2743	0.049*	
C15	−0.7400 (8)	0.4101 (3)	0.3976 (3)	0.0687 (9)	
H15A	−0.8667	0.3713	0.4372	0.103*	
H15B	−0.5491	0.3848	0.4169	0.103*	
H15C	−0.7986	0.4047	0.3159	0.103*	

### Atomic displacement parameters (Å^2^)

**Table:**

	*U*^11^	*U*^22^	*U*^33^	*U*^12^	*U*^13^	*U*^23^
O1	0.0495 (10)	0.0383 (11)	0.0708 (13)	0.0017 (8)	0.0294 (9)	−0.0014 (9)
O2	0.0604 (11)	0.0439 (11)	0.0593 (12)	−0.0043 (9)	0.0290 (10)	−0.0038 (9)
O3	0.0680 (13)	0.0610 (13)	0.0512 (12)	−0.0032 (10)	0.0335 (10)	0.0064 (9)
O1W	0.0532 (11)	0.0398 (10)	0.0783 (14)	−0.0022 (9)	0.0271 (10)	0.0019 (9)
N1	0.0682 (15)	0.0580 (15)	0.0502 (13)	0.0072 (13)	0.0343 (12)	−0.0005 (12)
N2	0.0362 (10)	0.0410 (11)	0.0421 (11)	0.0000 (10)	0.0183 (9)	−0.0052 (10)
N3	0.0316 (10)	0.0471 (12)	0.0378 (11)	0.0012 (9)	0.0138 (8)	−0.0056 (9)
C1	0.0305 (11)	0.0378 (13)	0.0363 (12)	−0.0033 (10)	0.0101 (9)	−0.0029 (10)
C2	0.0437 (13)	0.0367 (13)	0.0406 (13)	−0.0007 (11)	0.0199 (11)	0.0026 (10)
C3	0.0483 (14)	0.0364 (14)	0.0460 (14)	0.0041 (11)	0.0182 (12)	0.0000 (11)
C4	0.0390 (13)	0.0484 (15)	0.0385 (13)	−0.0013 (11)	0.0161 (11)	−0.0050 (11)
C5	0.0570 (15)	0.0480 (16)	0.0407 (14)	−0.0011 (13)	0.0227 (12)	0.0104 (12)
C6	0.0526 (15)	0.0372 (14)	0.0452 (14)	0.0025 (12)	0.0184 (12)	0.0028 (11)
C7	0.0282 (11)	0.0386 (14)	0.0421 (14)	−0.0011 (10)	0.0093 (10)	−0.0026 (11)
C8	0.0372 (12)	0.0430 (14)	0.0386 (13)	0.0039 (10)	0.0135 (10)	−0.0034 (10)
C9	0.0293 (11)	0.0459 (14)	0.0331 (12)	0.0025 (11)	0.0084 (9)	−0.0032 (11)
C10	0.0365 (13)	0.0452 (14)	0.0356 (12)	0.0012 (11)	0.0103 (10)	−0.0003 (11)
C11	0.0495 (15)	0.0501 (17)	0.0412 (14)	0.0055 (12)	0.0143 (12)	−0.0084 (12)
C12	0.0466 (14)	0.0589 (18)	0.0353 (13)	0.0068 (13)	0.0166 (11)	−0.0042 (13)
C13	0.0413 (14)	0.0544 (16)	0.0326 (13)	0.0031 (12)	0.0118 (11)	0.0027 (12)
C14	0.0422 (13)	0.0427 (14)	0.0391 (13)	0.0072 (11)	0.0148 (11)	−0.0014 (11)
C15	0.091 (2)	0.0530 (19)	0.070 (2)	−0.0032 (17)	0.0381 (19)	0.0106 (16)

### Geometric parameters (Å, º)

**Table:**

O1—C7	1.233 (3)	C3—H3A	0.9300
O2—C10	1.355 (3)	C4—C5	1.383 (4)
O2—H2	0.9261	C5—C6	1.379 (3)
O3—C13	1.377 (3)	C5—H5A	0.9300
O3—C15	1.413 (4)	C6—H6A	0.9300
O1W—H1W1	0.9247	C8—C9	1.459 (3)
O1W—H2W1	0.8339	C8—H8A	0.9300
N1—C4	1.383 (3)	C9—C10	1.387 (4)
N1—H1N1	0.9272	C9—C14	1.403 (4)
N1—H2N1	0.9864	C10—C11	1.398 (3)
N2—C7	1.353 (3)	C11—C12	1.366 (4)
N2—N3	1.376 (3)	C11—H11A	0.9300
N2—H2N	0.9473	C12—C13	1.386 (4)
N3—C8	1.284 (3)	C12—H12A	0.9300
C1—C2	1.387 (3)	C13—C14	1.381 (3)
C1—C6	1.390 (3)	C14—H14A	0.9300
C1—C7	1.478 (3)	C15—H15A	0.9600
C2—C3	1.370 (3)	C15—H15B	0.9600
C2—H2A	0.9300	C15—H15C	0.9600
C3—C4	1.396 (3)		
			
C10—O2—H2	110.2	O1—C7—C1	122.8 (2)
C13—O3—C15	117.1 (2)	N2—C7—C1	115.4 (2)
H1W1—O1W—H2W1	117.5	N3—C8—C9	121.5 (2)
C4—N1—H1N1	119.3	N3—C8—H8A	119.3
C4—N1—H2N1	110.4	C9—C8—H8A	119.3
H1N1—N1—H2N1	126.4	C10—C9—C14	119.5 (2)
C7—N2—N3	121.2 (2)	C10—C9—C8	122.5 (2)
C7—N2—H2N	124.2	C14—C9—C8	118.0 (2)
N3—N2—H2N	114.5	O2—C10—C9	122.7 (2)
C8—N3—N2	115.2 (2)	O2—C10—C11	118.1 (2)
C2—C1—C6	117.3 (2)	C9—C10—C11	119.2 (2)
C2—C1—C7	123.5 (2)	C12—C11—C10	120.8 (3)
C6—C1—C7	119.2 (2)	C12—C11—H11A	119.6
C3—C2—C1	121.7 (2)	C10—C11—H11A	119.6
C3—C2—H2A	119.1	C11—C12—C13	120.6 (2)
C1—C2—H2A	119.1	C11—C12—H12A	119.7
C2—C3—C4	120.7 (2)	C13—C12—H12A	119.7
C2—C3—H3A	119.7	O3—C13—C14	124.7 (3)
C4—C3—H3A	119.7	O3—C13—C12	115.8 (2)
C5—C4—N1	122.6 (2)	C14—C13—C12	119.5 (2)
C5—C4—C3	118.1 (2)	C13—C14—C9	120.5 (2)
N1—C4—C3	119.3 (2)	C13—C14—H14A	119.8
C6—C5—C4	120.8 (2)	C9—C14—H14A	119.8
C6—C5—H5A	119.6	O3—C15—H15A	109.5
C4—C5—H5A	119.6	O3—C15—H15B	109.5
C5—C6—C1	121.5 (2)	H15A—C15—H15B	109.5
C5—C6—H6A	119.3	O3—C15—H15C	109.5
C1—C6—H6A	119.3	H15A—C15—H15C	109.5
O1—C7—N2	121.8 (2)	H15B—C15—H15C	109.5
			
C7—N2—N3—C8	177.2 (2)	N3—C8—C9—C10	−2.5 (3)
C6—C1—C2—C3	1.3 (3)	N3—C8—C9—C14	177.0 (2)
C7—C1—C2—C3	−177.5 (2)	C14—C9—C10—O2	−179.8 (2)
C1—C2—C3—C4	−1.2 (4)	C8—C9—C10—O2	−0.3 (4)
C2—C3—C4—C5	0.2 (4)	C14—C9—C10—C11	0.9 (3)
C2—C3—C4—N1	−177.9 (2)	C8—C9—C10—C11	−179.6 (2)
N1—C4—C5—C6	178.6 (3)	O2—C10—C11—C12	179.8 (2)
C3—C4—C5—C6	0.6 (4)	C9—C10—C11—C12	−0.8 (4)
C4—C5—C6—C1	−0.5 (4)	C10—C11—C12—C13	−0.2 (4)
C2—C1—C6—C5	−0.4 (4)	C15—O3—C13—C14	−5.6 (4)
C7—C1—C6—C5	178.4 (2)	C15—O3—C13—C12	174.2 (3)
N3—N2—C7—O1	−0.8 (4)	C11—C12—C13—O3	−178.6 (2)
N3—N2—C7—C1	178.87 (19)	C11—C12—C13—C14	1.2 (4)
C2—C1—C7—O1	162.1 (2)	O3—C13—C14—C9	178.7 (2)
C6—C1—C7—O1	−16.6 (4)	C12—C13—C14—C9	−1.1 (4)
C2—C1—C7—N2	−17.6 (3)	C10—C9—C14—C13	0.0 (3)
C6—C1—C7—N2	163.7 (2)	C8—C9—C14—C13	−179.4 (2)
N2—N3—C8—C9	−179.7 (2)		

### Hydrogen-bond geometry (Å, º)

**Table:**

*D*—H···*A*	*D*—H	H···*A*	*D*···*A*	*D*—H···*A*
O1*W*—H1*W*1···O1^i^	0.92	2.00	2.926 (3)	174
O2—H2···N3	0.93	1.85	2.650 (3)	143
O1*W*—H2*W*1···O1^ii^	0.83	1.95	2.787 (3)	176
N2—H2*N*···O1*W*	0.95	2.15	3.084 (3)	167
N1—H1*N*1···O3^iii^	0.93	2.25	3.043 (3)	143
N1—H2*N*1···O2^i^	0.99	2.17	3.141 (3)	169
C2—H2*A*···O1*W*	0.93	2.45	3.351 (3)	163
C8—H8*A*···O1*W*	0.93	2.56	3.368 (3)	146

Symmetry codes: (i) −*x*+1, *y*−1/2, −*z*; (ii) −*x*, *y*−1/2, −*z*; (iii) *x*+2, *y*, *z*−1.
